# Aggregation of adult parasitic nematodes in sex-mixed groups analysed by transient anomalous diffusion formalism

**DOI:** 10.1098/rsif.2024.0327

**Published:** 2024-10-09

**Authors:** Ruth Leben, Sebastian Rausch, Laura Elomaa, Anja E. Hauser, Marie Weinhart, Sabine C. Fischer, Holger Stark, Susanne Hartmann, Raluca Niesner

**Affiliations:** ^1^ Institute for Immunology, Veterinary Medicine, Freie Universität Berlin, Berlin, Germany; ^2^ Dynamic and Functional in vivo Imaging, Veterinary Medicine, Freie Universität Berlin, Berlin, Germany; ^3^ Biophysical Analytics, Deutsches Rheuma-Forschungszentrum, A Leibniz Institute, Berlin, Germany; ^4^ Institute of Chemistry and Biochemistry, Freie Universität Berlin, Berlin, Germany; ^5^ Department of Rheumatology and Clinical Immunology, Immune Dynamics, Charité—Universitätsmedizin Berlin, Corporate Member of Freie Universität Berlin and Humboldt University, Berlin, Germany; ^6^ Laboratory for Immune Dynamics, Deutsches Rheuma-Forschungszentrum, A Leibniz Institute, Berlin, Germany; ^7^ Institute of Physical Chemistry and Electrochemistry, Leibniz Universität Hannover, Hannover, Germany; ^8^ Center for Computational and Theoretical Biology, Fakultät für Biologie, Julius-Maximilians-Universität Würzburg, Würzburg, Germany; ^9^ Institute of Theoretical Physics, Technische Universität Berlin, Berlin, Germany

**Keywords:** parasitic nematode, *Heligmosomoides bakeri*, movement, locomotion, transient anomalous diffusion, time-averaged mean square displacement

## Abstract

Intestinal parasitic worms are widespread throughout the world, causing chronic infections in humans and animals. However, very little is known about the locomotion of the worms in the host gut. We studied the movement of *Heligmosomoides bakeri,* naturally infecting mice, and used as an animal model for roundworm infections. We investigated the locomotion of *H. bakeri* in simplified environments mimicking key physical features of the intestinal lumen, i.e. medium viscosity and intestinal villi topology. We found that the motion sequence of these nematodes is non-periodic, but the migration could be described by transient anomalous diffusion. Aggregation as a result of biased, enhanced-diffusive locomotion of nematodes in sex-mixed groups was detected. This locomotion is probably stimulated by mating and reproduction, while single nematodes move randomly (diffusive). Natural physical obstacles such as high mucus-like viscosity or villi topology slowed down but did not entirely prevent nematode aggregation. Additionally, the mean displacement rate of nematodes in sex-mixed groups of 3.0 × 10^−3^ mm s^−1^ in a mucus-like medium is in good agreement with estimates of migration velocities of 10^−4^ to 10^−3^ mm s^−1^ in the gut. Our data indicate *H. bakeri* motion to be non-periodic and their migration random (diffusive-like), but triggerable by the presence of kin.

## 1. Introduction

One of the most common parasite infections are infections with parasitic worms also known as helminths. These parasites infect more than one-quarter of the world’s population with the roundworm *Ascaris lumbricoides* being one of the most prevalent human helminth infections globally [1]. More than 80% of the helminths dwell in the intestine of their hosts, in particular nematodes, the roundworms. The intestine is a very special environment to live in with exceptional physiological and physical characteristics: (i) a flow of mucus and ingested food (ingesta) in one direction; (ii) elasticity, it can stretch and contract; (iii) changes of its surface topology where long villi in the small intestine become shorter towards the large intestine, (iv) a viscoelastic mucus layer covering the luminal side, and (v) an environment in which millions of bacteria and possibly other pathogens (protozoa and fungi) coexist with the helminths [2]. Here nematodes reproduce and move; however, information about their locomotion is scarce.

The locomotion of parasitic nematodes differs from the locomotion of larger organisms, such as fish, and from the locomotion of micro-swimmers such as microscopic parasites (size in the micrometre range) [3], in terms of the forces involved, the average velocity and the resulting Reynolds number [4]. For instance, adult *Heligmosomoides bakeri,* previously named *Heligmosomoides polygyrus* [5,6]*,* in the following referred to as *H. bakeri*, a natural intestinal nematode of mice and model organism for experimental investigation of nematode infections, has a diameter of approximately 100 µm and a length of several millimetres to centimetres (sex-dependent) [7]. It typically appears in the coiled posture, whose diameters vary in the range of a few 100 µm. The adult worms can be found in the jejunum and distal duodenum of the small intestine during acute infection (14 days post-infection (p.i.)), but during chronicity can only be detected in the proximal part of the small intestine (21 days p.i.). Considering typical lengths of murine small intestine segments, we infer that the maximum displacement amounts to 10–15 cm within up to 7 days [8], resulting in an estimate for the displacement rate of 10^−4^ to 10^−3^ mm s^−1^.

As previously proposed for the free-living nematode *Caenorhabditis elegans*, nematode movement is assumed to be undulatory, being imposed by their vermiform structure with an interior segmented hydroskeleton, surrounded by contractile muscles and inextensible fibres of the exterior cuticle. The forces acting to result in the undulatory motion are alternative muscle contraction and relaxation in the different body segments and the interior pressure of the hydroskeleton, as previously modelled two dimensionally [9]. In addition, a three-dimensional helical motion has been shown for *Nippostrongylus brasiliensis*, however, only in viscous media, *in vitro*, with displacement supported by thrust in the viscous medium [10].

Using metabolic imaging based on NAD(P)H fluorescence lifetime imaging of *H. bakeri* in the infected mouse intestine, we could show that the adult worms adapt their bioenergetics to the high energy demand necessary for locomotion against the peristaltic flow, towards the upper part of the intestine [11]. Whether *H. bakeri* uses the two-dimensional undulatory or three-dimensional helical regimes to migrate in the intestine, whether this motion follows a periodic pattern, typical for multi-cellular organisms and how physical parameters, such as mucus viscosity and intestinal villi geometry influence the *H. bakeri* motion are still unknown. Additionally, the reasons for their migration towards the upper part of the duodenum remain elusive, posing the question whether they compete for scarce resources in the intestine, move randomly or cooperate.

Here, we isolated adult *H. bakeri* from the small intestine during 11–28 days p.i. and investigated their locomotion in different artificial environments. To reduce the complexity of the natural habitat, we separately addressed its individual characteristics, meaning the intestinal topology, the medium viscosity or the presence of other individuals of the same species. We found that the motion sequence of these nematodes is non-periodic. Their trajectories can be analysed by relying on the anomalous diffusion theory, previously used to analyse particle and cell motion among others [12,13], but taking into account transient motion regime. We show that transient anomalous diffusion describes *H. bakeri* migration, resulting in the aggregation of worms in sex-mixed groups.

## 2. Material and methods

### 2.1. *Heligmosomoides bakeri* infection

Wild-type C57BL/6 mice aged 8−10 weeks were purchased from Janvier Labs (Saint-Berthevin, France). All animals were maintained under specific pathogen-free (SPF) conditions and were fed standard chow ad libitum. Mice were infected with 200 third-stage infective (L3) *H. bakeri* larvae via oral gavage in 200 μl drinking water. On the dissection days, the mice were sedated by inhalation of isofluorane and sacrificed by cervical dislocation, followed by extraction of the small intestine. Individual female and male *H. bakeri* worms were isolated from the small intestines of infected mice and were plated out in a 96-well plate containing approximately 150 μl/well of RPMI medium. The worms were incubated in serum-free RPMI with 2 × PS at 37°C for 24 h to remove remains of mouse tissue, mucus and microbial contaminations prior to imaging experiments. All animal experiments were performed in accordance with the National Animal Protection Guidelines and approved by the German Animal Ethics Committee for the Protection of Animals in Berlin (LAGeSO, G0207/19).

### 2.2. Microscopic and mesoscopic imaging

Microscopic images were taken with the head of a commercial laser scanning microscope (TriMScope II, LaVision Biotec, a Miltenyi company, Bielefeld, Germany) used in transmitted light, wide field mode. The transmitted light of a white light lamp was collected by an objective (UPlan FLN, 4×, NA 0.13, Olympus, Hamburg, Germany) and detected by a commercial 18.0 megapixel digital reflex camera (EOS600D, Canon, Japan) attached to one of the oculars of the microscope by a specialized adapter (Canon EF-M-Kamerabajonett for LM Digital SLR Adapter, Micro Tech Lab, Graz, Austria) instead of the camera objective. The movies were recorded in time-lapse mode with 25 frames s^−1^. The image dimensions/pixel size was determined by the grid spacing (63.5 µm) of a square pattern 400-mesh copper grid (3.05 mm diameter, TEM support grid, Agar Scientific, Stansted, Essex, UK).

Mesoscopic imaging was carried out with a standard stereoscope (Hund, Wetzlar, Germany) used in transmitted light mode. The transmitted light of a white light lamp was collected by an objective (0.64× or 0.8× magnification) and detected the same way as the microscopic images, by the Canon digital reflex camera attached to the stereoscope ocular with the specialized adapter. The movies were recorded in time-lapse mode with 1 frame s^−1^. The image dimensions/pixel size was determined by the diameter (3.05 mm) or the grid spacing (63.5 µm) of the square pattern 400-mesh copper grid.

### 2.3. Nematode handling and movement analysis conditions

*Heligmosomoides bakeri* are exothermic organisms, meaning they stop moving when the temperature of the environment is too cold. For imaging, a heating plate with a hole for light transmission was placed under the plates with the *H. bakeri* worms to keep the medium at 31°C.

For microscopic imaging, the worms were placed individually in a 96-well plate with a U-shaped bottom to keep the worms in focus to study the motion patterns as well as to create ‘constrained conditions’ of the movement.

For mesoscopic imaging, the worms were placed individually or in sex-mixed cohorts of 5–10 animals in a flat-bottomed 24-well plate with (14 mm diameter) to create movement conditions with constraints at much larger scales than the nematode dimensions, similar to the situation in the host gut, i.e. ‘quasi-free’ movement conditions on a flat plate.

The worms were kept in an RPMI cell culture medium to provide them with nutrients, and when not imaged, they were held in a 37°C incubator. For imaging, the worms were gently placed on the well plates using disposable transfer pipettes with the tips cut off to increase their diameter.

### 2.4. SYTOX Green staining

For live/dead staining, 100 µl SYTOX^®^ Green staining (Thermo Fisher Scientific, Darmstadt, Germany) was added in approximately 500 µl worm suspensions. SYTOX^®^ Green is a green fluorescence nucleic acid stain that does not penetrate living cells and, by that, is an indicator of dead cells or, in our case, small dead organisms. The fluorophore was excited with a mercury vapour lamp, the emission was detected as described above for microscopic imaging after passing a 534/40 nm bandpass filter (AHF analysentechnik AG, Tübingen, Germany).

### 2.5. Alginate mixture and viscosity measurement

To create mucus-like viscosities, the cell culture medium RPMI was thickened with 2% sodium alginate, following published research [10]. Sodium alginate is not toxic as it is used in food processing technology and forms a transparent gel in the presence of calcium ions. The resulting viscosity of 2% alginate measured by rotation viscometry (ATAGO VISCO-895, Gimat GmbH Liquid Monitoring, Polling, Germany) is approximately 77 mPa s. This value is similar to that of mucus in the small intestine [14]. To determine the viscosity and shear-thinning properties of the alginate mixture, we used an A1 spindle (diameter 17 mm) together with a small beaker (diameter 22 mm, resulting fluid height 30 mm), as well as an adapter for low viscosities (spindle diameter 25 mm, beaker diameter 26 mm, fluid height 91 mm). The rotational speed was varied between 30 and 250 rounds min^−1^ for the A1 spindle, and between 10 and 60 rounds min^−1^ for the low viscosity set-up. We calculated the shear forces in order to harmonize the viscosities measured with the two test set-ups (electronic supplementary material, S2).

### 2.6. Three-dimensional printed villi scaffolds to mimic intestinal topology

A porcine small intestine, 28 mm in diameter, was procured from a local meat wholesale market (Rasch, Berlin, Germany) and subjected to decellularization following an established protocol with slight modifications [15,16]. The mucosa and serosa were mechanically removed, and the intestine was subsequently sectioned into smaller pieces and immersed in 1% sodium dodecyl sulphate (Sigma Aldrich) for 2 h. Following chemical decellularization at room temperature, the tissue pieces were rinsed and incubated with phosphate buffered saline (PBS) for an additional hour. Subsequently, they were treated with fresh 1% gentamycin/PBS solution for five cycles, concluding with immersion in PBS. The resulting decellularized small intestine submucosa (dSIS) was further processed through enzymatic digestion and chemical functionalization for application in three-dimensional printing. For enzymatic digestion, lyophilized dSIS was exposed to an acidic pepsin solution (100 mg of pepsin and 1 g of dry dSIS in 100 ml of 0.01 N HCl, pH 2) at room temperature for two days. Pepsin digestion was terminated by adjusting the pH to 9 with 1 N NaOH solution. The digested dSIS was then methacryloylated by sequentially adding methacrylic anhydride (MAAH) six times into the solution over 3 h (totalling 1 ml of MAAH per 1 g of dSIS), while maintaining the pH at 9 with 1 N NaOH. Subsequently, the pH was adjusted to 7.4, and the resulting dSIS-MA polymer was dialysed against PBS for 1 day followed by distilled water for 6 days before lyophilization. To formulate the dSIS-MA into a bioactive three-dimensional printing resin, it was dissolved in 0.01 N NaOH at a concentration of 1.5 wt%, along with 1 wt% of polyethylene glycol dimethacrylate (4 kDa, Sigma Aldrich) and 1 wt% of lithium phenyl−2,4,6-trimethylbenzoylphosphinate photoinitiator (Sigma Aldrich). Intestine-mimicking three-dimensional hydrogel scaffolds (diameter of 11 mm, villi diameter of 300–500 µm and variable height from 0.5 to 2 mm) were designed using Rhinoceros 5 software and fabricated with a visible light DLP printer (Photon D2, Anycubic), employing a 45 s cross-linking time for every 100 µm layer. Following three-dimensional printing, non-cross-linked macromers and dye residues were removed from the samples by excessive immersion in milli-Q water at room temperature.

### 2.7. Image analysis: pre-processing and object tracking

To analyse nematode movement from the acquired time-lapse videos, we first needed to pre-process the data. Using Fiji ImageJ (v. 1.54g [17]), the standard deviation image of the videos was subtracted from the raw data to remove the image background and to retain only the changing/moving parts in the movies. The RGB videos were transformed to 8-bit grey value images and colour-inverted for tracking. The full dynamic range of intensity (8-bit) was used for all images, for image standardization. Tracking was performed by TrackMate an ImageJ plug-in [18] and verified by visual inspection. We used the threshold detector to identify and segment single nematodes in the individual images and a basic linear assignment problem solver (LAP) for object (nematode) tracking in the videos. In case the automatic tracking was erroneous, we manually connected tracks belonging to the same nematode or separated tracks when two nematodes overlapped. The object tracking results were exported and transferred to a custom-written Python script to create plots of nematode trajectories, all with the starting point at the origin (0,0), i.e. rose plots, as well as to calculate the time-averaged mean-squared displacements (MSD). Relying on the MSD time dependence, we analysed and represented the directionality of nematode movement (see §3). The exponent of the MSD time dependence was determined by linear regression (Origin2023, OriginLab, CA). To determine the dependence of the exponent α on the time scale τ, we determined the exponent for MSD segments containing five data points each.

**Figure 1.**
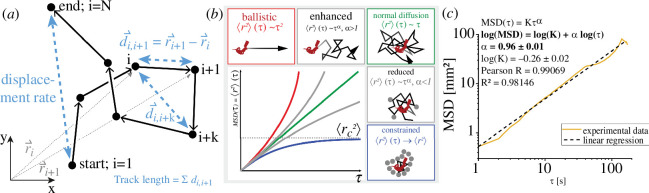
Anomalous diffusion theory: motion regimes determined by the time dependence of the time-averaged mean square displacement MSD(τ). (*a*) Sketch of relevant vectors and distances to determine MSD(τ). These also form the basis of the different speed measures as well as the confinement ratio and linearity of forward progressions. Track displacement is the ‘air line’ from the starting point to the end point of the track. Total distance travelled is the sum of all distances *d*_*i*,*i *+1_ between time points *i* and *i*+1, *r*_*i*_ is the position at time *i*. Graphic adapted from [15]. (*b*) Movement categories according to the diffusion theory: clockwise from upper left to bottom right: ballistic movement (α = 2, red); enhanced-diffusive movement (α > 1); normal diffusion (α = 1, green); reduced-diffusive movement (α < 1); movement under constrained condition (α = 0, blue). Graphic adapted from [19]. (*c*) Representative linear fit (black, dashed line) of an experimental MSD time dependence (orange curve) in double-logarithmic representation, used to determine the slope α, i.e. the exponent in non-logarithmic representation equation (3.2).

From each of the tracks, TrackMate determines the track mean speed, the displacement rate and the resulting linearity of forward progressions as well as the confinement ratio. The *track mean speed* is the average of all instantaneous velocities of the track, $track\;mean\;speed=\frac{track\;length}{track\;duration}$. The *mean displacement rate* is the net displacement (air line between the start and end of the track), divided by the track duration (see figure 1*a*): $displacement\;rate=\frac{track\;displacement\;}{track\;duration}$. The *linearity of forward progression* is defined as the ratio between the displacement rate and the track mean speed: $linearity\;of\;forward\;progression=\frac{displacement\;rate}{track\;mean\;speed}$. The *confinement ratio* is the ratio of track displacement divided by the track length and indicates how efficiently the nematode migrates. It is a unitless measure, taking values between 0 and 1, 0 indicating a fully confined movement and 1 meaning directed movement along a straight line: $confinement\;ratio=\frac{track\;displacement}{track\;length}$ [15].

## 3. Results

### 3.1. The body posture of *H. bakeri* at rest is adapted to the intestinal topology

**Figure 2.**
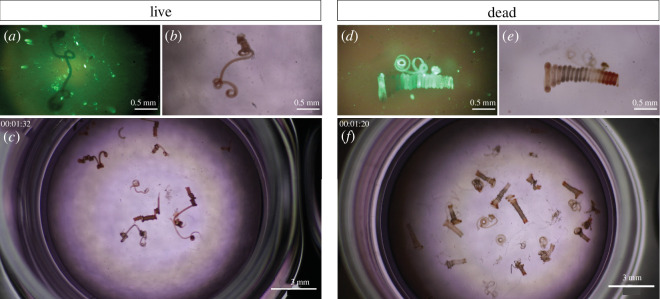
Minimum energy requirement of *H. bakeri* in tightly coiled, helical posture. Comparison of living (left) versus dead (right) *H. bakeri* worms in the cell culture medium heated to 31°C. Top row: Microscopic images at 4 × magnification; (*a*) and (*d*) SYTOX^®^ Green fluorescence indicating dead cells/organism. (*b*) and (*e*) Transmitted light microscopy of the same individuals a few seconds later than (*a*) and (*d*). Scale bars indicate 0.5 mm. Bottom rows (*c*) and (*f*) show still images of mesoscopic movies at 0.67× magnification (electronic supplementary material, video S1). Scale bars indicate 3 mm. All dead individuals are tightly coiled, in contrast to actively stretching live nematodes.

By comparing the posture of live and dead nematodes in the cell culture medium, we found that a coiled geometry of the worm is the one associated with a minimum energy requirement, as is the posture of all dead worms (figure 2 and electronic supplementary material, video S1). Live nematodes stretch parts of their body to move and eventually to travel. Labelling with SYTOX Green, a dye that intercalates with the DNA of dead cells, demonstrated the death of the nematodes. The nematodes were killed either by low (−20°C) or high temperature (+100°C), or chemically (4% Neopredisan), to exclude the specific impact of physical or chemical conditions on the muscle structure, which defines the body posture.

We found that the loops of *H. bakeri* at rest are 295 ± 10 µm in diameter (mean value ± s.d., 11 individuals) for both females and males, with the longer females building more loops than the shorter males [7]. The loop diameter at rest is slightly smaller than the typical diameter of a villus in the duodenum of mice, determined by two-photon microendoscopy (300 ± 19 µm, electronic supplementary material, figure S1).

### 3.2. Non-periodic *H. bakeri* motion can be described by transient anomalous diffusion

**Figure 3.**
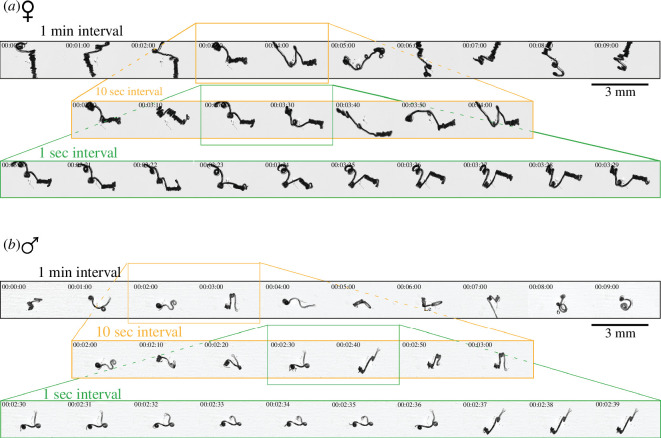
Non-periodic motion patterns of *H. bakeri*. (*a*) and (*b*) Sequences of still images of microscopic movies (electronic supplementary material, video S2) at 4× magnification of adult female (*a*) and male (*b*) *H. bakeri* worms in the cell culture medium heated to 31°C. Sequences have different temporal intervals: top row (black frame)—10 min sequence with 1 min interval time; middle row (orange frame)—1 min zoom-in sequence with 10 s interval time; bottom row (green frame)—10 s zoom-in sequence with 1 s interval time (1 Hz). The time shown is min:s. Scale bars indicate 0.5 mm and apply for all shown sequences. The movement is irregular, meaning no motion pattern is repeated, e.g. in a sinusoidal (two-dimensional) or helical (three-dimensional) movement.

The sequence of still images and the temporal zoom-in from electronic supplementary material, video S2 (figure 3) reveal that the motion of *H. bakeri* is a series of apparently random stretching and coiling that do not result in a closed and periodic motion cycle. Hence, in contrast to other nematodes, such as *C. elegans* or *N. brasiliensis*, *H. bakeri*, does not move using periodic patterns, typical for multi-cellular organisms, i.e. neither a two-dimensional undulatory nor a three-dimensional helical motion.

Thus, no clear periodic pattern of the motion sequence of either female or male worms could be identified. The time-dependent instantaneous velocity of representative male and female nematode trajectories (shown in figure 3) did not show any patterns (electronic supplementary material, figure S3).

In the next step, we aimed to study the movement of nematodes in sex-mixed groups under intestine-like physical conditions. We hypothesize that the motion of *H. bakeri* can be described by anomalous diffusion. The diffusion theory describes stochastic molecular motion (Brownian motion), following Fick’s laws. It has been extended with the concept of anomalous diffusion, to describe the motion of molecules, but also of cells in tissues [12,13] or of uni- and multi-cellular organisms [20,21], in the presence of additional conditions, such as confinements or different types of transport processes. The time-averaged MSD is a measure of the distance that an object travels on average in the lag time τ=k ×Δt [22] and is defined as


(3.1) $$MSD\left(\tau \right)=\;\frac{1}{N-k}\sum_{i=1}^{N-k}{\left|{\vec{r}}_{i+k}-{\vec{r}}_{i}\right|}^{2},$$

where $\vec{r_{i}}$ (with i=1,…,N-k denoting the successive frames of a track and with index k=1,…,N-1) is the position of the object (worm) at the *i*th time point, ${\vec{r}}_{i+k}$ is the position of the object at the (*i *+ *k*)th time point, delayed by τ=k ×Δt , and ∆t the constant time span between two successive frames (figure *1a*).

Following the anomalous diffusion theory, the time dependence of the time-averaged MSD of an object is generally described by


(3.2) $$MSD\left(\tau \right)=K\;\times \tau^{\alpha },$$

with *K* being a constant with different meanings for the different motion regimes, τ being the lag time and α being the exponent, which describes the motion regimes. For infinite and continuous times as well as an infinite space, the following five motion regimes can be identified by α:

— α = 0: confinement/constrained conditions, such as motion but no displacement, for which MSD approaches a constant over time,
— 0 < α < 1: sub-diffusive regime, such as random motion with obstacles,
— α = 1: diffusive motion, stochastic motion following Fick’s laws, such as Brownian motion,
— 1 < α < 2: super-diffusive regime, meaning an oriented motion, as an overlapping directed and random motion, such as the one resulting from Levy walks [23,24], and
— α = 2: ballistic motion, such as straight directed locomotion.

In contrast to theory, the experimental time spans are finite and discrete and the space is limited. The following observations should not be termed as ‘anomalous diffusion’ [23], but rather ‘transient anomalous diffusion’. Thus, the sub-diffusive regime characterizes a reduced-diffusive motion and the super-diffusive regime an enhanced diffusion regime due to biased motion, which on different time scales may change, having a transient character.

First, we verified whether the (transient) anomalous diffusion formalism also applies to *H. bakeri* nematode motion. Nematode motion in U-shaped dishes serves as a proof-of-principle for confinement, in contrast to random free nematode motion on flat dishes, both in aqueous medium (electronic supplementary material, video S2 and electronic supplementary material, video S3).

**Figure 4.**
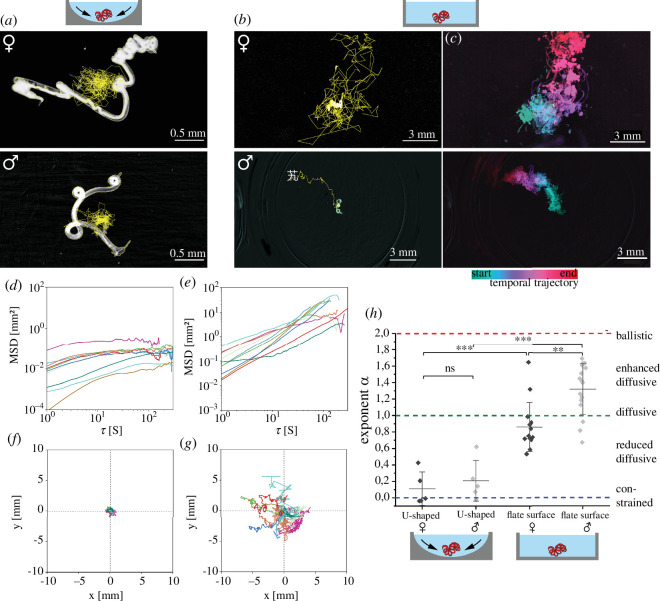
Analysis of nematode motion regimes under confined and quasi-free-movement conditions. Single adult *H. bakeri* worms in the aqueous cell culture medium heated to 31°C; the top row shows exemplary images of a female worm, the bottom row of a male worm (one of five per sex and condition). (*a*) The representative microscopic image of one worm (of five) of each sex under constrained conditions in a dish with a U-shaped bottom with 4× magnification, scale bars are 0.5 mm. (*b*) Motion of one worm (of five) of each sex on a flat bottom plate; 4× magnification (electronic supplementary material, video S3), scale bars indicate 3 mm. Yellow lines in (*a*) and (*b*) show nematode trajectories (tracking analysis by TrackMate). (*c*) Colour-coded temporal trajectories of the data shown in (*b*). (*d*) and (*e*) Time-averaged mean square displacement (MSD) of all tracks: (*d*) corresponds to motion under confined conditions, (*e*) and to random, free motion on a flat plate. (*f*) and (*g*) To (*d*) and (*e*) corresponding rose plots, i.e. plots of nematode trajectories, with the starting point set to the origin (0,0). (*h*) Exponent α resulting from the linear fit of the double-logarithmic time-dependent MSD, displayed per sex and condition. α indicates the motion regime, according to the anomalous diffusion theory. Statistical significance is represented by ***p ≤ 0.001; **p ≤ 0.01; *p ≤ 0.05; *p *> 0.05: ns, using the one-way ANOVA with Bonferroni’s post hoc test (U-shape: n_male = 5, n_female = 5; flat-surface: n_male = 11, n_female = 10). Under unconfined conditions, the non-periodic motion of *H. bakeri* results in a forward movement.

By comparing the time dependence of MSD of both single male and female nematodes under partially confined conditions, i.e. when placed on a U-shaped well in aqueous media, and to their free movement on a flat well, much larger than their size, we found that the theory of anomalous diffusion reliably describes the nematode motion for the given time window (figure 4*a,d,f*).

The exponent α resulting from the linear regression of the time-dependent MSD for partially confined nematode motion is 0.16 ± 0.11 (*n* = 10 worms, i.e. 5 males and 5 females), with no difference between males and females (figure 4*a,d,f*). Moreover, the corresponding rose plots (figure 4*f*) displaying the nematode trajectories ($\vec{r}-{\vec{r}}_{0}$) indicate no displacement for all individuals. Electronic supplementary material, figure S3 presents instantaneous velocity, rose plot, MSD, exponent α over lag time τ of the female and male worm shown in figure 4*a* and electronic supplementary material, video S2.

In contrast, for freely moving nematodes on a flat plate the exponent α increases significantly to 1.10 ± 0.08 (*n* = 21 worms, i.e. 11 males and 10 females), indicating a diffusive motion regime (figure 4*b,c*,*e*,*g*), resembling random motion, similar to the stochastic Brownian motion of molecules at thermic equilibrium. However, sex-dependent analysis of the MSD reveals a shift towards the reduced regime for females (α = 0.86 ± 0.07) and the enhanced-diffusive regime for males (α = 1.32 ± 0.08). Additionally, the freely moving nematode trajectories show displacement, but no preferential orientation of locomotion (rose plots in figure 4*g*).

### 3.3. Enhanced-diffusive motion of *H. bakeri* in sex-mixed cohorts leads to worm aggregation, despite physical intestinal obstacles

**Figure 5.**
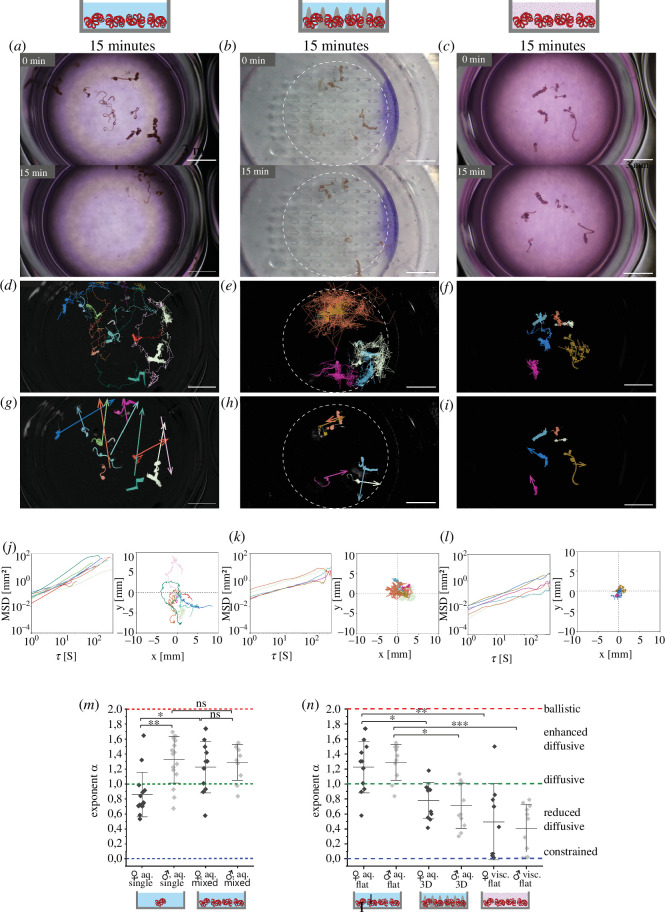
Enhanced-diffusive, biased motion of *H. bakeri* in sex-mixed cohorts indicates aggregation, being subject to physical obstacles in the intestine. Analysis of the movement of adult *H. bakeri* worms of both sexes in cohorts and in different environments, at 31°C, observed over 15 min: (left—*a*,*d*,*g*,*j*) in an aqueous cell culture medium on a flat plate; (middle—*b*,*e*,*h*,*k*) in an aqueous culture medium on a three-dimensional printed villi structure, dashed circle indicates the area with printed villi; (right—*c*,*f*,*i*,*l*) in a viscous cell culture medium thickened with 2% alginate on a flat plate. (*a–c*) Still images of mesoscopic movie at 0.67× magnification (electronic supplementary material, video S4). Scale bars indicate 3 mm. (*d–f*) Still images of processed data shown in (*a–c*) with segmented and tracked worms (TrackMate), entire trajectories are shown. (*g–i*) Still images of processed data are shown in (*a–c*) with the displacement vectors of segmented and tracked worms from (*d–f*). (*j–l*) Time-averaged mean square displacement (MSD) of the tracks (left) and corresponding rose plots (right). (*m*) and (*n*) Exponent α of the fit of the MSDs per sex under the above presented conditions. (*m*) Single male and female worms versus female and male worms in a cohort on a flat plate in an aqueous medium (single: n_male = 11, n_female = 10; mixed: n_male = 10, n_female = 11). (*n*) Female and male worms in a cohort: left—on a flat plate in an aqueous medium, middle—in an aqueous culture medium on the three-dimensional printed villi structure, right—in the cell culture medium thickened with 2% alginate on a flat plate (flat_mixed: n_male = 10, n_female = 11; three-dimensional print: n_male = 11, n_female = 11; viscous: n_male = 11, n_female = 9). Statistical significance is represented by ***p ≤ 0.001; **p ≤ 0.01; *p ≤ 0.05; *p *> 0.05: ns, using one-way ANOVA with Bonferroni’s post hoc test.

In order to investigate the interaction between nematodes of different sexes and the impact on their locomotion, we compared the motion of nematodes in sex-mixed cohorts on the flat plate, in the aqueous medium (figure 5*a,d*,*g,j*; each group with *n* = 5 to 6 females and *n* = 5 to 6 males) to the motion of single nematodes, under the same conditions. We found an increase of the exponent α from 1.01 ± 0.14 (diffusive regime) to 1.26 ± 0.15 (enhanced diffusive), for *n* = 21 worms (figure 5*m*). Additionally, all nematodes migrated to the same area 4 min after being randomly placed on the well surface (electronic supplementary material, video S4). These observations hint towards an aggregation of the worms. When characterizing the motion regimes in a sex-dependent manner, we found that the reduced motion regime of single females (α = 0.86 ± 0.07, *n* = 10) turns into an enhanced-diffusive regime of females in sex-mixed groups (α = 1.22 ± 0.17, *n* = 11). In contrast, the enhanced-diffusive motion regime of single males (α = 1.32 ± 0.08, *n* = 11) is preserved also in sex-mixed groups (α = 1.29 ± 0.12, *n* = 10). This may indicate that females rather than males concur with the aggregation.

The natural habitat of *H. bakeri* is the small intestine, the luminal-sided surface of which has a villi structure, which are protrusions of the *lamina propria mucosae*. These villi increase the surface area of the intestine for efficient nutrient absorption from the ingesta. We mimicked the intestinal topology with three-dimensional printed scaffolds of extracellular matrix-based material (villus diameter 300 µm, gradient in the height 0.5 to 2.0 mm), to investigate its influence on the worm movement. We found that this topology impedes the nematode motion in sex-mixed groups (figure 5*b*,*e*,*h*,*k*), independent of the villus length, as we detected a reduced-diffusive regime of nematode motion in sex-mixed cohorts when placed on the three-dimensional scaffolds in aqueous media, with α = 0.74 ± 0.13 (*n* = 10 worms, 11 males and 11 females, figure 5*n*), within 15 min observation time.

The *lamina propria mucosae* is covered with a layer of viscoelastic mucus, the viscosity of which is 3 mPa s < η < 100 mPa s (depending on the layer depth [14]) and, thus, higher than the aqueous medium (η ≃ 1 mPa s). As we expected the viscoelastic mucus to impact on the nematode movement, we imitated the viscosity of intestinal mucus by 2% alginate in the aqueous medium, as previously reported for *N. brasiliensis* movement investigations [10]. The viscosity η of 2% alginate in the cell culture medium ranges from approximately 80 mPa s to approximately 60 mPa s measured by rotation viscometry. Similar to intestinal mucus, 2% alginate has viscoelastic properties, as it is a shear-thinning fluid (electronic supplementary material, S2). However, the 2% alginate medium used in our experiments is a homogeneous viscoelastic mixture, whereas the rheology of the intestinal mucus is far more complex, related to spatial zonation [25].

We analysed the motion of sex-mixed cohorts on a flat well, in this viscous (mucus-like) medium and found that it impedes the nematode motion as well (figure 5*c*,*f*,*i*,*l*). Again, it resembles reduced-diffusive features (α = 0.45 ± 0.10, *n* = 20 worms, 11 males and 9 females) within 15 min observation time (figure 5*n*).

For both physically impeding conditions, i.e. the villi-like scaffolds and mucus-like viscous medium, we found no significant differences between male and female motion regimes in sex-mixed cohorts (figure 5*n*).

**Figure 6.**
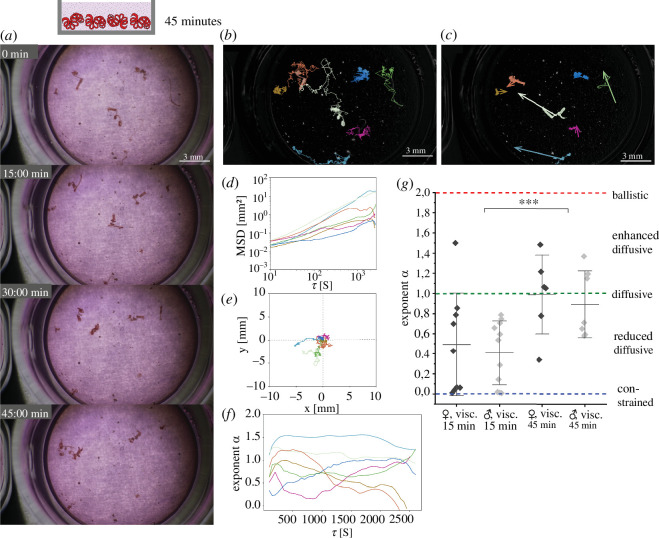
Increasing orientation of nematode movement on larger time scales, despite obstacles of mucus-like environment. Analysis of the movement of adult *H. bakeri* worms of both sexes in a cohort in the viscous cell culture medium thickened with 2% alginate on a flat plate heated to 31°C. (*a*) Still images of 45 min mesoscopic movie at 0.67× magnification (electronic supplementary material, video S5). (*b*) The still image of processed data shown in (*a*) with segmented and tracked worms (by TrackMate); entire tracks are presented. (*c*) Still image of processed data shown in (*a*), with displacement vectors of segmented and tracked worms (*b*). Scale bars in (*a*), (*b*) and (*c*) indicate 3 mm. (*d*) Time-averaged mean squared displacement (MSD) of the tracks and (*e*) corresponding rose plots. (*f*) Dependence of exponent α of the fit of the MSDs on the lag-time τ for the tracks shown in (*d*) and (*e*). (*g*) Exponent α of the fit of the MSDs per sex under the above presented conditions: left, within 15 min and right, within 45 min (15 min: n_male = 11, n_female = 9; 45 min: n_male = 7, n_female = 6). Statistical significance is calculated for the entire cohorts (not separated according to sex) and represented by ***p ≤ 0.001, Student’s *t*‐test. Individual worms are able to overcome the obstacles of viscosity within 45 min and migrate towards each other.

Notably, we found that the worms in sex-mixed cohorts in the viscous medium (2% alginate) do migrate towards each other as well, when we observed them over longer periods of time, i.e. 45 min (figure 6). However, their motion in the viscous medium is slower than in the aqueous medium, with a mean displacement rate of 0.003 mm s^−1^ compared with 0.03 mm s^−1^ (15 min in the aqueous medium). Additionally, we found an increase of exponent α to 0.94 ± 0.17 (*n* = 13 worms, 7 males and 6 females), when analysing the time dependence of MSD for over 45 min (at 0.1 Hz image acquisition rate) instead of 15 min (at 1 Hz acquisition rate). The nematode motion regime cannot be described exclusively as sub-diffusive, diffusive or super-diffusive, as (following the anomalous diffusion theory) this would mean that the regimes do not change on different time scales. It is rather described by transient reduced diffusion, changing towards diffusion or even biased, enhanced diffusion. The transient character of the reduced-diffusive motion regime is emphasized by the dependence of the exponent α on the time scale, indicated by τ (figure 6*f*). The exponent α increases for short time scales for most worms, to a value above 1, indicating an enhanced-diffusive regime, for longer time scales. These observations are supported by the steeper MSD(τ) curves of the first tracked 15 min as compared with the next 30 min (15 to 45 min time window) shown in electronic supplementary material, S5, with the instantaneous velocity of the nematodes not changing in the respective time windows (electronic supplementary material, S5).

Altogether, these results indicate that nematodes can overcome physical intestinal-like obstacles to aggregate and the reduced-diffusive migration is transient towards increasing orientation over longer observation times.

### 3.4. Supportive characteristic numbers of *H. bakeri* movement

To consolidate the results obtained by the MSD analysis presented above, we compared the track mean speed, i.e. the average instantaneous speed of a track, the mean displacement rate and the resulting linearity of forward progressions as well as the confinement ratio for all investigated conditions (table 1).

**Table 1.** Track mean speeds, displacement rate, confinement ratios and linearity of forward progressions for all conditions.

		track mean speed (mm s^−1^)	displacement rate (mm s^−1^)	confinement ratio	linearity of forward progression
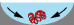	**single worms, U-shaped plate, aqueous medium**	0.09 ± 0.03	0.0008 ± 0.0005	0.0098 ± 0.006	0.011 ± 0.006
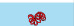	**single worms, flat plate, aqueous medium**	0.18 ± 0.13	0.02 ± 0.01	0.15 ± 0.13	0.17 ± 0.13
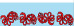	**sex-mixed, flat plate, aqueous medium**	0.14 ± 0.05	0.03 ± 0.02	0.3 ± 0.2	0.23 ± 0.19
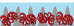	**sex-mixed,** **three-dimensional print, aqueous medium**	0.2 ± 0.1	0.004 ± 0.002	0.03 ± 0.05	0.03 ± 0.02
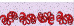	**sex-mixed, flat plate, viscous medium**	0.064 ± 0.063	0.001 ± 0.001	0.035 ± 0.042	0.036 ± 0.042

As shown in table 1, we found a high variability of nematode motion under the same experimental conditions (electronic supplementary material, S4). However, the mean displacement rate values show that the worms are several orders of magnitude quicker when moving quasi-freely, i.e. in an aqueous medium, on a flat plate, as compared with physical obstacles, i.e. when placed on the three-dimensional printed villi scaffolds or in a mucus-like viscous medium. In contrast, the track mean speed stays the same within the error margins for worms moving in an aqueous medium, both on flat plates and on three-dimensional scaffolds, but it decreases in a viscous medium. The confinement ratio and linearity of forward progression values impeded the constrained nematode movement of three-dimensional scaffolds in a viscous medium.

As the forward movement depends on the type of fluid flow (laminar or turbulent) at the nematode–medium interface, we additionally investigated the range of Reynolds number values for *H. bakeri*. The Reynolds number Re is defined as


(3.3) $$Re=\frac{v\;\tilde{L}\;\rho }{\eta },$$

where v is the velocity of the swimmer, $\tilde{L}$ is the characteristic length (volume of the swimmer divided by the area of the surface facing the direction of travel), ρ is the density of the fluid, and η is the dynamic viscosity of the fluid.

In the case of *H. bakeri,* the motion sequence is non-periodic; thus the characteristic length continuously changes during motion and is also subject to inter-individual variations as previously demonstrated and discussed. For this reason, the Reynolds number of *H. bakeri* is not a constant number but varies within a range. In an aqueous medium, the Reynolds number ranges from Re = 11 for a completely stretched nematode to Re = 0.7 for a completely coiled nematode. However, these extremes are never reached under real conditions, because both body postures do not result in an effective forward motion. In a 2% alginate medium, the Reynolds number range is 0.003–0.014, implying a laminar flow of the medium at the interface with the nematode. As the 2% alginate mixture, similar to intestinal mucus, is a viscoelastic, shear-thinning fluid, the range may change depending on the specific motion sequence of the nematodes.

## 4. Discussion

Gastrointestinal nematodes account for the vast majority (about 80%) of parasitic worm infections, affecting approximately a quarter of the world’s population, in particular, in tropical and sub-tropical areas as well as farm and wild animals all over the world. These infections are of considerable global concern leading to millions of disability-adjusted life years of infected humans and to huge economic losses in animal husbandry. *Heligmosomoides bakeri,* naturally infecting mice, represents an easily accessible mouse model to study parasitic nematode infections. Briefly, after oral uptake, the larvae invade the small intestinal submucosa before re-emerging and dwelling in the gut lumen as long-lived adult worms, leading to chronic infections. Previous studies mostly focused on the persistence mechanisms of parasitic nematodes and their efficient immunomodulation resulting in chronic worm persistence in immunocompetent hosts. This included the immunoregulation of innate host immune cells [26–28], as well as adaptive immune responses [29–32] and the significant regulation of unrelated autoimmune responses [33–36]. Moreover, nematodes induce epithelial immunological quiescence at the transcriptional level [37], alter the composition of the microbiota and release antimicrobial factors [2,38,39], even promoting the production of antimicrobials by host cells, potentially thwarting efficient anti-parasite immune responses [38]. However, despite the close coexistence of intestinal nematodes with millions of bacteria in a mucus-rich environment, induced by the infection itself [2,40], little or nothing is known about the locomotion of adult worms in the gut, allowing them not only to persist but also to mate and reproduce in the specific host niche, i.e. the small intestine. In particular, the impact of physical conditions such as mucus viscosity and luminal topology and of nematode coexistence, i.e. social condition, on their locomotion is not clear.

By analysing the posture (geometry) of nematodes as compared with the intestinal topology, consisting of periodically organized villi, we found that the coiled body of nematodes at rest, with no energetic needs, is adapted to the villus geometry in the duodenum. We suggest that this adaptation ensures nematode attachment to the villi and, by that, resistance against the peristaltic flow in the host intestine. The coiled geometry continuously changes in live, motile nematodes, however, not following any obvious periodic pattern, as expected from other nematodes (*C. elegans* [41,42] or *N. brasiliensis* [10]). Relying on the anatomical fact that longitudinal muscles surround the tubular body of the nematodes directly under the cuticula, we infer that these muscles have a helical geometry at rest, which leads to the coiled body posture, and that the worms need to actively stretch against this internal tension. Some of these muscles contract locally, inducing stretching of the neighbouring, connected muscles. This differs from *C. elegans*, for which an alternate contraction of the muscles on transversally opposite sides of the body, leads to a two-dimensional undulatory motion.

To be able to determine the effects of physical cues, i.e. medium viscosity and environment topology, and social cues, in sex-mixed groups, on nematode locomotion, we investigated nematode motion in environments with simple geometry in which these cues could be individually controlled. The observed motion does not resemble the complexity of nematode movement in the mouse intestine but provides us with insight into its specific aspects.

By analysing the time-averaged MSD of nematode trajectories moving under confined conditions as compared with those freely moving on a flat surface, we found that the anomalous diffusion theory applies to their non-periodic movement. Relying on this theory, we could show that freely moving nematodes in sex-mixed groups can be described by an enhanced-diffusive regime, in contrast to single nematodes, with differences between sexes. As we found that randomly distributed nematodes on a flat surface migrate to the same spot (aggregate), we infer that the enhanced-diffusive motion is of cooperative, social nature, but not collective (swarm-like) as their motions are not synchronized. With ‘cooperative’ we mean not competitive. This behaviour is possibly triggered by mating and reproduction, similar to the effect of pheromones in *N. brasiliensis* [43]. Notably, we observed a similar aggregation also for other nematodes, i.e. the human and porcine intestinal nematode *Ascaris suum* (unpublished data), indicating that this may represent an overarching nematode adaptation. Next to social cues (mating), we expect also chemical gradients in the host intestine to impact nematode migration and lead to nematode aggregation, as it has been shown that adult males migrate faster than adult females towards the upper duodenum when placed at the same (distal) site in the host small intestine [44].

Moreover, we found that higher medium viscosity, similar to that of mucus, and intestinal-like villi topology, lead to reduced-diffusive nematode motion in sex-mixed groups, suggesting that these physical cues represent motion obstacles. However, these physical obstacles can be overcome, as we found the orientation of locomotion when extending the observation time window of nematodes in a mucus-like viscous medium, with similar shear-thinning properties.

As we observed that nematodes attempt to coil around the three-dimensional printed villi, sporadically attaching to those for short periods of time (electronic supplementary material, video S6), the reduced-diffusive nematode motion may also be explained by short time spans, during which the nematodes pause and inspect the villus structure. In contrast to intestinal villi, on the acellularized three-dimensional printed villi structure we did not observe long-term nematode attachment. Possibly, this is due to insufficient adhesion of the nematodes on the smooth surface of the three-dimensional-printed villi compared with that of natural cellularized intestinal villi, as microvilli and glycocalyx on epithelial cells lining the villus create a rough surface at the nanometre scale [45]. The presence of epithelial cells on the villi may also be required for long-term nematode attachment as the parasite feeds on these host cells and not on other nutrient sources such as ingesta or blood [46].

Notably, the migratory velocity, i.e. displacement rate (3 × 10^−3^ mm s^−1^), of nematodes in a viscous mucus-like medium is in good agreement with the migratory velocity of 10^−4^ to 10^−3^ mm s^−1^ we estimated for worms travelling maximum several tenths of centimetres through the intestine, over a time span of several days, starting from the time point the adult worms emerge from the submucosa into the lumen and ending at the time point when they arrive at the upper part of the duodenum.

In conclusion, by analysing nematode motion relying on the anomalous diffusion theory, we found that nematodes migrate in an oriented manner to aggregate, in sex-mixed groups, suggesting adaptation triggered by mating and reproduction, with high mucus-like viscosity and intestinal villi topology, slowing down, but not preventing the nematodes from gathering.

## Data Availability

Raw data are available on Zenodo under [47]. Supplementary material is available online [48].
